# Burnout and stress: new insights and interventions

**DOI:** 10.1038/s41598-025-92909-6

**Published:** 2025-03-11

**Authors:** Yi-lang Tang, Antonino Raffone, Samuel Yeung Shan Wong

**Affiliations:** 1 Department of Psychiatry and Behavioral Sciences, Emory University, Atlanta, GA 30329 USA; 2 Substance Abuse Treatment Program, Maxwell Cleland Atlanta VA Medical Center, Decatur, GA 30033 USA; 3 Department of Psychology, Sapienza University of Rome, Piazzale Aldo Moro 5, 00185 Rome, Italy; 4 JC School of Public Health and Primary Care, The Chinese University of Hong Kong, Hong Kong Special Administrative Region, China

**Keywords:** Burnout, Stress, Resiliency, Systemic factors, Stress management, Interventions, Health care, Comorbidities

## Abstract

Burnout, characterized by emotional exhaustion, depersonalization, and reduced personal accomplishment, has become increasingly prevalent in modern society, particularly among healthcare professionals. The chronic stress experienced in these demanding roles significantly contributes to the development of burnout. This editorial reviews recent research findings on burnout and stress, focusing on findings from *Scientific Reports*' Collection on the topic. We explore the role of both systemic factors and individual vulnerabilities in contributing to burnout across various contexts, from healthcare settings to academia. Recent studies highlight the role of protective psychological factors such as optimism, humor, and resilience in mitigating burnout, while also emphasizing how hope and self-efficacy can mediate the relationship between stress and professional burnout. Moral injury and systemic inefficiencies faced by healthcare professionals were exacerbated during the COVID-19 pandemic. Analysis of cognitive-behavioral stress-management competencies reveals that proactive approaches, particularly planning and prevention, are more effective than reactive methods in managing stress. The evidence suggests that effective interventions must address both systemic issues (such as excessive workloads and resource constraints) and individual factors (through resilience-building and stress-management training). A holistic approach combining institutional support with individual empowerment strategies is essential for mitigating burnout and stress and enhancing collective well-being in professional settings.

Burnout is a psychological syndrome characterized by a self-reported state of care- or work-related physical and mental stress, manifesting as emotional exhaustion (EE), depersonalization (DP), and a sense of reduced personal accomplishment (PA)^1^. This condition has emerged as a defining symptom of modern life, exacerbated by the increasingly fast-paced, high-stress environments prevalent in today’s society. The COVID-19 pandemic has starkly highlighted and intensified these challenges, leading to a surge of burnout and stress across various professions, including healthcare professionals and those working in academia. In response to this critical issue, *Scientific Reports* launched a timely Collection on Burnout and Stress in 2023. This editorial aims to explore recent research on burnout and stress, with a special emphasis on findings from the Collection. We will focus primarily on healthcare professionals, who have been at the forefront of the pandemic response and have experienced unprecedented levels of stress and burnout^2^. Additionally, we will underscore the urgent need for both systemic and individualized approaches to mitigate the impact of burnout.

## The multifaceted nature of burnout and stress and their broad implications

Burnout and stress, while often linked to work, might stem from both systemic factors and individual vulnerabilities. Studies such as Enav et al.^3^ highlight protective factors like optimism, humor, and resilience that mitigate burnout in parenting contexts, even during crises like the pandemic. Farhi and Rubinsten^4^ found that teachers’ stress levels predicted their well-being, with the relationship mediated by their difficulty in emotional regulation. These findings underscore the interplay of personal traits and external stressors in shaping the trajectories of burnout and stress.

Similarly, Szczęśniak et al.^5^ explored how psychological constructs like hope and self-efficacy mediate the relationship between stress and professional burnout among corporate employees. Their findings highlight that fostering hope—defined as the motivation and pathways to achieve goals—can buffer stress and reduce burnout. Such insights point to the value of psychological interventions in building resilience against chronic occupational stress.

Importantly, burnout and stress are not merely a personal affliction; they may have far-reaching consequences for families, organizations, and communities. Parental burnout, for instance, negatively impacts child well-being and family dynamics^3^. In healthcare, burnout can compromise patient care and exacerbate workforce shortages^6^. The ripple effects of burnout demand coordinated action across policy, organizational, and individual levels.

## Burnout among healthcare professionals

Healthcare professionals face unique stressors that render them particularly vulnerable to burnout. These include long hours, poor work-life balance, perceived sense of lack of support or reward^6–9^, and systemic inefficiencies such as staff shortages, heavy patient load, and administrative burdens^6^. The COVID-19 pandemic further exacerbated these pressures, introducing additional challenges like moral injury and resource constraints.

A study by Usset et al.^10^ on moral injury among healthcare professionals found that experiences incongruent with personal and professional values significantly contributed to burnout and turnover intentions. For example, situations where healthcare professionals were forced to ration care due to resource shortages heightened emotional exhaustion and depersonalization. Such findings emphasize the need for systemic interventions to address value misalignments and moral distress in healthcare settings.

The National Academy of Medicine’s 2019 report on clinician burnout and well-being highlighted systemic problems like high workloads, administrative burdens, and inadequate staffing as key contributors to burnout. However, most interventions have focused on individual resilience rather than systemic changes^6^. This imbalance underscores the importance of addressing organizational and structural factors to create supportive work environments.

## Cognitive-behavioral competencies in stress management

The study by Epstein et al.^11^ offers valuable insights into the relative importance of cognitive-behavioral stress-management competencies. The researchers identified four key competencies: (1) managing or reducing sources of stress: Identifying and mitigating stressors; (2) Managing Thoughts: Using cognitive strategies to reframe stress-inducing situations; (3) Planning and Prevention: Proactively organizing tasks to avoid stress; (4) Practicing Relaxation Techniques: Employing methods like mindfulness or breathing exercises to manage stress.

The study, which analyzed data from over 18,000 participants across 125 countries, found that proactive stress-management practices—particularly planning and prevention—were more effective than reactive methods. Regression analyses indicated that these competencies were strongly associated with self-reported happiness and success, both personal and professional. The findings suggest that equipping individuals with proactive stress-management skills can significantly improve their resilience and overall well-being.

For healthcare professionals, these competencies can be particularly impactful. Planning and prevention could involve scheduling breaks to avoid fatigue, while relaxation techniques could help mitigate the acute stress of high-stakes situations. Institutions should support these practices by offering training programs that emphasize proactive stress management.

## Burnout in crisis contexts

Extreme conditions like wars and pandemics bring unique stressors that intensify burnout. Tsybuliak et al.’s study^12^ on Ukrainian academic staff during the ongoing conflict revealed rising levels of emotional exhaustion and depersonalization. Factors such as university relocation and professional instability disproportionately affected women, emphasizing the gendered dimensions of burnout. These findings call for targeted interventions to support vulnerable subgroups within stressed populations.

In healthcare, moral injury—the distress experienced when one’s actions conflict with deeply held values—has emerged as a critical contributor to burnout. Usset et al.^10^ demonstrated the downstream effects of exposure to morally injurious events on healthcare workers’ turnover intentions and burnout. Their research underscores the urgent need for systemic reforms to address moral distress, such as aligning organizational values with those of workers and reducing administrative burdens.

## Towards effective interventions

The complexities of factors associated with burnout and stress necessitate a multifaceted response. Recent findings suggest both systemic and individual strategies can mitigate burnout and stress effectively.

Addressing systemic issues such as excessive workloads, lack of resources, and misaligned organizational values can significantly alleviate occupational stress. For instance, Usset et al.^10^ highlighted the importance of flexible scheduling and value alignment in reducing burnout among healthcare workers. Institutional support, such as psychological counseling and crisis management, is vital in extreme contexts, as evidenced by the heightened burnout levels among Ukrainian academics^12^. Farhi and Rubinsten^4^ also emphasized the importance of providing teachers with tools to manage their emotions effectively in the classroom to reduce stress and burnout. Promoting personal resilience through psychological constructs like optimism and humor can buffer stress and enhance well-being. Enav et al.’s study^3^ suggests training programs to cultivate these traits could benefit parents facing high stress. Encouraging hope and self-efficacy, as explored by Szczęśniak et al.^5^ offers a pathway to bolster employees’ capacities to cope with stress and achieve personal and professional goals. Leveraging Epstein et al.’s findings^11^ on cognitive-behavioral competencies, training programs can focus on proactive stress-management techniques like planning and cognitive reframing to empower individuals.

Additionally, integrating systemic and personal strategies can provide a holistic solution. For example, pairing flexible policies with resilience-building workshops can create a supportive environment that nurtures individual well-being.

## Conclusions

Burnout and stress among professionals require urgent attention. Insights from recent studies underscore the importance of addressing both systemic barriers and individual vulnerabilities. By fostering supportive environments and promoting resilience, we can mitigate the adverse effects of burnout and enhance collective well-being. Cognitive-behavioral competencies, especially proactive stress-management techniques, offer a promising avenue for intervention. As we move forward, let us prioritize sustainable work practices and holistic strategies that empower individuals and institutions to thrive amidst challenges.
